# Supplementary material for: Screening of cytoprotectors against methotrexate-induced cytogenotoxicity from bioactive phytochemicals
Source: PeerJ. 2016 May 11;4:e1983. doi: 10.7717/peerj.1983 (PMC4867711; doi:10.7717/peerj.1983)
Supplement: Data S1 [file peerj-04-1983-s001.doc]

Induction ratio data of *umu* gene by MTX (50 μg ml^-1^) in *Salmonella typhimurium* TA1535/pSK1002 with bioactive phytochemicals treatment

| Extract | Extracts Effective Components  Concentration (mg ml^-1^) | R value | | |
| --- | --- | --- | --- | --- |
|  |  | Ⅰ | Ⅱ | Ⅲ |
| control | 0 | 8.59 | 9.85 | 10.12 |
| Chlorogenic Acid (Chrysanthemum) | 0.0001 | 8.48 | 9.24 | 9.61 |
|  | 0.001 | 8.28 | 9.3 | 9.48 |
|  | 0.01 | 8.51 | 8.74 | 9.45 |
|  | 0.05 | 8.91 | 9.18 | 9.90 |
|  | 0.25 | 8.28 | 9.33 | 8.40 |
| Allicin  (Garlic) | 0.001 | 7.09 | 6.95 | 7.95 |
|  | 0.01 | 5.61 | 6.23 | 6.85 |
|  | 0.05 | 6.44 | 5.13 | 5.98 |
|  | 0.25 | 5.97 | 5.16 | 4.95 |
|  | 1.25 | 4.17 | 5.59 | 4.76 |
| Gingerols  (Ginger root) | 0.001 | 8.77 | 9.23 | 9.81 |
|  | 0.01 | 9.67 | 10.53 | 9.23 |
|  | 0.1 | 8.45 | 8.96 | 9.53 |
|  | 1 | 8.37 | 9.28 | 9.47 |
|  | 5 | 8.36 | 9.50 | 8.72 |
| Ginkgo flavone  (Ginkgo leaf) | 0.01 | 8.37 | 9.41 | 9.07 |
|  | 0.1 | 9.92 | 9.1 | 9.27 |
|  | 1 | 8.77 | 9.12 | 9.74 |
|  | 5 | 8.12 | 9.23 | 8.39 |
|  | 25 | 8.23 | 7.27 | 7.12 |
| Ginsenosides  (Ginseng root) | 0.01 | 10.3 | 9.61 | 9.37 |
|  | 0.1 | 10.42 | 9.75 | 9.29 |
|  | 0.5 | 8.33 | 9.23 | 8.21 |
|  | 2.5 | 8.36 | 8.61 | 9.31 |
|  | 12.5 | 8.64 | 8.65 | 9.62 |
| Proanthocyanidins  (Grape seed) | 0.001 | 8.59 | 9.85 | 10.12 |
|  | 0.01 | 9.49 | 8.14 | 8.56 |
|  | 0.1 | 8.12 | 7.08 | 7.51 |
|  | 1 | 7.11 | 6.17 | 6.22 |
|  | 5 | 5.12 | 6.34 | 5.82 |
| Polyphenols  (Green tea) | 0.01 | 8.75 | 9.01 | 9.72 |
|  | 0.1 | 8.51 | 8.13 | 7.42 |
|  | 1 | 6.93 | 6.24 | 7.11 |
|  | 10 | 5.11 | 5.43 | 6.02 |
|  | 50 | 3.74 | 3.97 | 4.98 |
| Polysaccharides  (Reishi mushroom) | 0.001 | 9.54 | 10.3 | 10.8 |
|  | 0.01 | 9.98 | 8.92 | 9.15 |
|  | 0.1 | 8.26 | 8.91 | 9.20 |
|  | 1 | 8.22 | 8.85 | 7.89 |
|  | 5 | 7.69 | 6.92 | 6.57 |
| Eleutherosides  (Siberian Ginseng root) | 0.001 | 8.94 | 7.45 | 8.48 |
|  | 0.01 | 7.26 | 6.82 | 8.21 |
|  | 0.1 | 4.37 | 5.53 | 4.68 |
|  | 0.5 | 3.21 | 3.83 | 4.75 |
|  | 1.5 | 3.76 | 2.14 | 3.49 |
| Isoflavones  (Soybean) | 0.001 | 8.85 | 8.22 | 9.66 |
|  | 0.01 | 8.11 | 8.93 | 7.53 |
|  | 0.1 | 8.04 | 6.92 | 7.33 |
|  | 1 | 7.48 | 6.62 | 6.45 |
|  | 5 | 7.1 | 6.18 | 5.56 |

Induction ratio data of *umu* gene by MTX (50 μg ml^-1^) in *Salmonella typhimurium* TA1535/pSK1002 with bioactive phytochemical combinations

| Treatment | R value | | |
| --- | --- | --- | --- |
|  | Ⅰ | Ⅱ | Ⅲ |
| A | 4.65 | 4.84 | 5.55 |
| B | 5.84 | 7.33 | 6.34 |
| C | 6.73 | 7.14 | 6.47 |
| D | 2.51 | 4.46 | 3.28 |
| E | 6.06 | 7.75 | 6.74 |
| AB | 3.58 | 5.44 | 3.94 |
| AC | 4.75 | 5.43 | 4.36 |
| AD | 3.37 | 2.67 | 3.89 |
| AE | 4.53 | 4.12 | 5.21 |
| BC | 5.14 | 5.96 | 4.38 |
| BD | 2.1 | 2.01 | 3.12 |
| BE | 5.12 | 5.31 | 6.83 |
| CD | 2.68 | 2.22 | 3.14 |
| CE | 5.07 | 6.46 | 5.36 |
| DE | 3.66 | 2.54 | 3.16 |
| Control | 11.32 | 7.99 | 9.25 |

Note: A: Garlic allicin, B: Grape seed proanthocyanidins, C: Green tea polyphenols, D: Siberian ginseng Eleutherosides, E: Soybean isoflavones, AB: Garlic allicin + Grape seed proanthocyanidins; AC: Garlic allicin + Green tea polyphenols, AD: Garlic allicin + Siberian ginseng eleutherosides, AE: Allicin + Soybean isoflavones, BC: Grape seed proanthocyanidins + Green tea polyphenols, BD: Grape seed proanthocyanidins + Siberian ginseng eleutherosides, BE: Grape seed proanthocyanidins + Soybean isoflavones, CD: Green tea polyphenols + Siberian ginseng eleutherosides, CE: Green tea polyphenols + Soybean isoflavones, DE: Siberian ginseng eleutherosides + Soybean isoflavones. The concentration of each bioactive phytochemical was 1mg ml^-1^.

Number of PCE and NCE of Kunmin mice

| Groups | Number of mice treated/sex | Number of PCE observed | | | | | Number of PCE with MN | | | | | Number of MN | | | | | Number of NCE | | | | |
| --- | --- | --- | --- | --- | --- | --- | --- | --- | --- | --- | --- | --- | --- | --- | --- | --- | --- | --- | --- | --- | --- |
|  |  | Ⅰ | Ⅱ | Ⅲ | Ⅳ | Total | Ⅰ | Ⅱ | Ⅲ | Ⅳ | Total | Ⅰ | Ⅱ | Ⅲ | Ⅳ | Total | Ⅰ | Ⅱ | Ⅲ | Ⅳ | Total |
| Control | 4 F | 1512 | 1585 | 1584 | 1565 | 6246 | 4 | 3 | 5 | 4 | 16 | 4 | 3 | 5 | 4 | 16 | 1811 | 1975 | 1473 | 1592 | 6851 |
|  | 4 M | 1511 | 1539 | 1532 | 1516 | 6098 | 6 | 7 | 7 | 5 | 25 | 6 | 8 | 7 | 6 | 27 | 1788 | 1895 | 1334 | 1996 | 7013 |
| Model | 4 F | 1532 | 1548 | 1525 | 1553 | 6158 | 22 | 33 | 24 | 30 | 109 | 24 | 34 | 27 | 30 | 115 | 2897 | 2082 | 2966 | 1885 | 9830 |
|  | 4 M | 1562 | 1541 | 1522 | 1583 | 6212 | 25 | 37 | 28 | 34 | 124 | 27 | 38 | 30 | 36 | 131 | 3347 | 2512 | 2120 | 2913 | 10892 |
| TreatmentⅠ | 4 F | 1561 | 1527 | 1575 | 1513 | 6176 | 16 | 24 | 17 | 22 | 79 | 16 | 24 | 19 | 23 | 82 | 2301 | 1658 | 1814 | 2285 | 8058 |
|  | 4 M | 1504 | 1565 | 1513 | 1556 | 6138 | 25 | 16 | 18 | 22 | 81 | 26 | 18 | 19 | 23 | 86 | 2605 | 1687 | 2317 | 1815 | 8424 |
| TreatmentⅡ | 4 F | 1515 | 1546 | 1521 | 1561 | 6143 | 12 | 17 | 11 | 18 | 58 | 13 | 17 | 14 | 19 | 63 | 1782 | 2349 | 1628 | 1997 | 7756 |
|  | 4M | 1549 | 1507 | 1495 | 1561 | 6112 | 13 | 20 | 18 | 14 | 65 | 14 | 22 | 19 | 14 | 69 | 2461 | 1708 | 1812 | 1922 | 7903 |

Note: 1) Control Group = saline; Model Group = MTX +normal saline; Treatment GroupⅠ= MTX + combination of green tea polyphenols and eleutherosides from Siberian ginseng; Treatment GroupⅡ= MTX + combination of grape seed proanthocyanidins and eleutherosides from Siberian ginseng; 2)F = female mice; M = male mice.

The weight of mice body and their immune organs

| Groups | Number of mice treated /sex | body weight (g) | | | | Thymus weight(mg) | | | | Spleen weight (mg) | | | |
| --- | --- | --- | --- | --- | --- | --- | --- | --- | --- | --- | --- | --- | --- |
|  |  | Ⅰ | Ⅱ | Ⅲ | Ⅳ | Ⅰ | Ⅱ | Ⅲ | Ⅳ | Ⅰ | Ⅱ | Ⅲ | Ⅳ |
| Control | 4 F | 33.15 | 31.2 | 33.31 | 34.56 | 121.33 | 81.74 | 94.27 | 137.55 | 139.89 | 161.62 | 131.57 | 148.26 |
|  | 4 M | 30.24 | 32.18 | 35.12 | 32.14 | 96.77 | 138.7 | 83.23 | 91.28 | 133.66 | 176.67 | 142.94 | 145.92 |
| Model | 4 F | 31.21 | 29.15 | 34.26 | 31.26 | 38.39 | 71.71 | 24.32 | 32.2 | 65.23 | 53.64 | 74.00 | 90.97 |
|  | 4 M | 33.44 | 30.16 | 28.35 | 33.14 | 46.48 | 71.78 | 37.14 | 35.79 | 64.54 | 88.67 | 45.93 | 65.95 |
| TreatmentⅠ | 4 F | 29.15 | 31.45 | 34.26 | 33.18 | 50.43 | 66.36 | 95.93 | 61.05 | 106.11 | 102.84 | 155.54 | 131.06 |
|  | 4 M | 34.67 | 32.54 | 31.35 | 35.24 | 59.29 | 68.98 | 92.8 | 80.7 | 136.95 | 121.05 | 126.65 | 162.46 |
| TreatmentⅡ | 4 F | 30.84 | 28.86 | 33.21 | 34.61 | 85.74 | 120.35 | 76.72 | 105.91 | 115.65 | 140.26 | 130.52 | 116.98 |
|  | 4M | 32.63 | 33.44 | 35.34 | 31.42 | 89.41 | 131.08 | 85.88 | 86.41 | 128.56 | 181.91 | 122.28 | 129.45 |

Note: 1) Control Group = saline; Model Group = MTX +normal saline; Treatment GroupⅠ= MTX + combination of green tea polyphenols and eleutherosides from Siberian ginseng; Treatment GroupⅡ= MTX + combination of grape seed proanthocyanidins and eleutherosides from Siberian ginseng; 2)F = female mice; M = male mice.

Sperm head abnormalities of male Kunmin mice

| Groups | | Total number of sperm observed | Sperms with abnormal head | Constituent of different sperma with abnormal head morphology | | | |
| --- | --- | --- | --- | --- | --- | --- | --- |
|  |  |  |  | Lack hook | Amorphous | Banana-like | triangular |
| Control | Ⅰ | 1000 | 16 | 8 | 11 | 2 | 1 |
|  | Ⅱ | 1000 | 26 | 6 | 9 | 1 | 0 |
|  | Ⅲ | 1000 | 21 | 11 | 10 | 3 | 2 |
|  | Ⅳ | 1000 | 17 | 7 | 8 | 2 | 1 |
|  | Ⅴ | 1000 | 27 | 9 | 13 | 4 | 1 |
|  | Ⅵ | 1000 | 21 | 7 | 12 | 1 | 1 |
|  | total | 6000 | 128 | 48 | 63 | 11 | 6 |
| Model | Ⅰ | 1000 | 59 | 24 | 24 | 2 | 0 |
|  | Ⅱ | 1000 | 41 | 15 | 28 | 3 | 1 |
|  | Ⅲ | 1000 | 42 | 18 | 21 | 1 | 1 |
|  | Ⅳ | 1000 | 44 | 22 | 25 | 3 | 1 |
|  | Ⅴ | 1000 | 50 | 19 | 27 | 4 | 0 |
|  | Ⅵ | 1000 | 47 | 22 | 19 | 2 | 1 |
|  | total | 6000 | 283 | 120 | 144 | 15 | 4 |
| TreatmentⅠ | Ⅰ | 1000 | 22 | 12 | 14 | 3 | 0 |
|  | Ⅱ | 1000 | 34 | 8 | 12 | 2 | 2 |
|  | Ⅲ | 1000 | 26 | 10 | 15 | 4 | 0 |
|  | Ⅳ | 1000 | 33 | 12 | 13 | 2 | 1 |
|  | Ⅴ | 1000 | 25 | 9 | 10 | 3 | 1 |
|  | Ⅵ | 1000 | 23 | 14 | 13 | 2 | 1 |
|  | total | 6000 | 163 | 65 | 77 | 16 | 5 |
| TreatmentⅡ | Ⅰ | 1000 | 24 | 11 | 14 | 2 | 1 |
|  | Ⅱ | 1000 | 31 | 9 | 12 | 4 | 2 |
|  | Ⅲ | 1000 | 21 | 10 | 15 | 2 | 1 |
|  | Ⅳ | 1000 | 24 | 8 | 11 | 3 | 2 |
|  | Ⅴ | 1000 | 30 | 9 | 13 | 3 | 0 |
|  | Ⅵ | 1000 | 26 | 10 | 11 | 2 | 1 |
|  | total | 6000 | 156 | 57 | 76 | 16 | 7 |

Note: 1) Control Group = saline; Model Group = MTX +normal saline; Treatment GroupⅠ= MTX + combination of green tea polyphenols and eleutherosides from Siberian ginseng; Treatment GroupⅡ= MTX + combination of grape seed proanthocyanidins and eleutherosides from Siberian ginseng.
